# Editorial

**Published:** 1986-02

**Authors:** 


### Editorial — February 1986

Nobody reads Editorials, but every editor hopes that his may be the exception – and perhaps this may be one of them, because for most people receiving this number, it will have arrived 'out of the blue', unexpected and uninvited. Some explanation is needed. About 5 years ago Dr Ian McKee, a practising Edinburgh G. P., started a local Journal which he called EDINBURGH MEDICINE. He achieved a large circulation by distributing it free to all the doctors in the area and was thus able to attract enough advertising to pay for it. He set up a publishing company – Hermiston Publications and repeated his success by starting a similar journal in Glasgow – GLASGOW MEDICINE. This was followed by SCOTTISH MEDICINE for the rest of Scotland and then by MANCHESTER MEDICINE. Encouraged by the success of his new movement for the re-establishment of local journals, most of which had in the past become defunct, he turned to our area as a distinct geographical entity, and found that there was already a local journal in existence, but with a circulation too small to pay for itself by advertising. He therefore approached us and invited us to associate with him in his movement. This we are glad to do because it widens our horizon and relieves our small society of a financial burden. And so – here is the explanation of this manifestation, and of our additional title BRISTOL MEDICINE. Dr McKee started his EDINBURGH MEDICINE 'for the fun of it', and that's what keeps the rest of us at it. But he brought something else to the enterprise that the rest of us did not have – courage and business acumen. This is a precarious business and profits on one journal may be needed to balance losses on another; moreover changes in Government policy towards advertising may kill the enterprise at any time. Dr McKee deserves our congratulations and thanks.

The BRISTOL MEDICO-CHIRURGICAL JOURNAL was founded by the Bristol Medico-Chirurgical Society in 1883 and is the oldest local medical journal in this country – if not in the world. In 1953 it had ambitions to become a regional journal and renamed itself the MEDICAL JOURNAL OF THE SOUTH WEST. The South West however took little interest, the new name provided indexing problems and in 1963 it resumed its original title. The policy of the journal has always been to print scientific articles, especially by local authors. This gives an opportunity to our up-and-coming young doctors (even when students, see p. 3) to get their work into print and into the Index Medicus. For some years now we have been printing Abstracts of papers given at West of England Specialist Societies and have been building up an archive of value to the whole of the South West. We have a panel of correspondents who report on matters of interest in their spheres or on the world in general. Additional correspondents are being recruited from the West of England, and we welcome Dr A. E. Adam from Taunton. We also aim to provide reading of general interest to doctors, as for example Foreign Travel and leisure pursuits. We hope always to be able to give you something to read with pleasure and usually also with profit, and that you will also write to us with news and views and send us your articles. Remember that, through us, you can reach all your colleagues in the South West.

We shall arrive through your letter box every other month. The Nov/Dec. number will contain an Index. If you wish to keep the Journal for reference purposes the plastic binding spines available for a few pence from most stationers will hold the 6 numbers for a year. If our fate is to be your wastepaper basket we hope that you will have at least paused a moment and turned our pages before depositing us there.

